# Bis(2-amino­thia­zole-4-acetato)aquazinc(II)

**DOI:** 10.1107/S1600536809045589

**Published:** 2009-11-04

**Authors:** Lai-Jun Zhang, Xing-Can Shen, Yan Yang, Hong Liang

**Affiliations:** a Department of Chemistry, Shangrao Normal University, Shangrao 334001, People’s Republic of China; b Key Laboratory for the Chemistry and Molecular Engineering of Medicinal Resources (Ministry of Education), School of Chemistry and Chemical Engineering, Guangxi Normal University, Guilin 541004, People’s Republic of China; c Department of Chemistry and Biology, Yulin Teachers’ College, Yulin 537000, People’s Republic of China

## Abstract

In the title compound, [Zn(C_5_H_5_N_2_O_2_S)_2_(H_2_O)], the central Zn atom (2 site symmetry) is five-coordinated by two N and three O atoms [Zn—N = 2.047 (3) Å, Zn—O = 2.099 (2) and 1.974 (4) Å] in a distorted square-pyramidal geometry. Besides one O atom from a water mol­ecule, two 2-amino­thia­zole-4-acetate ligands provide two N and two O atoms as coordinated atoms. In the crystal structure, inter­molecular O—H⋯O and N—H⋯O hydrogen bonds connect the mol­ecules into an infinite three-dimensional framework.

## Related literature

For the pharmacological activity of potential metal-based drugs consisting of the thia­zole ligands and some physiologically active metal ions, see: Addison *et al.* (1984 ▶); Bolos *et al.* (1999 ▶); Chang *et al.* (1982 ▶); Dea *et al.* (2008 ▶). For related structures, see: Zhang *et al.* (2008*a* ▶,*b* ▶); Sen *et al.* (1997 ▶).
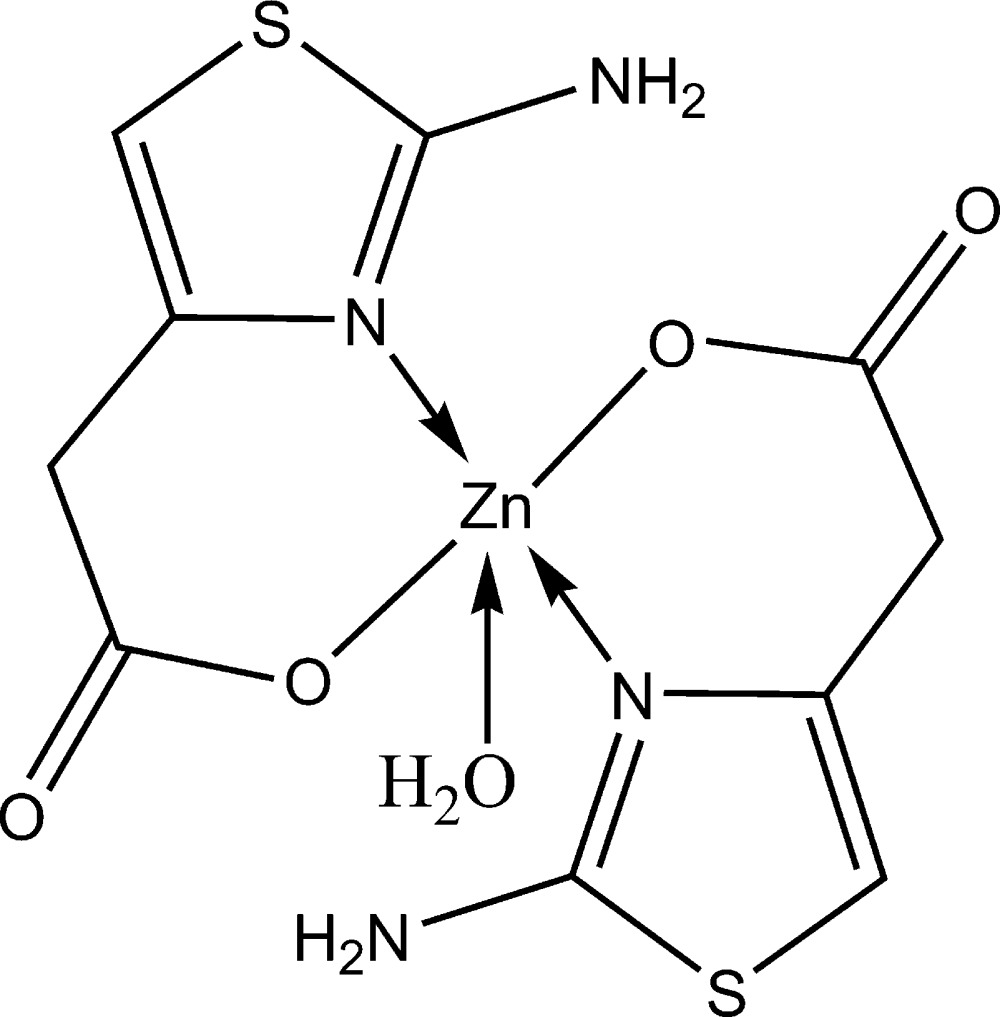


## Experimental

### 

#### Crystal data

[Zn(C_5_H_5_N_2_O_2_S)_2_(H_2_O)]
*M* *_r_* = 397.77
Monoclinic, 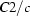

*a* = 11.715 (2) Å
*b* = 9.822 (2) Å
*c* = 12.580 (3) Å
β = 91.24 (3)°
*V* = 1447.2 (5) Å^3^
*Z* = 4
Mo *K*α radiation
μ = 2.01 mm^−1^
*T* = 295 K
0.12 × 0.10 × 0.08 mm

#### Data collection

Bruker APEXII CCD area-detector diffractometer
Absorption correction: multi-scan (*SADABS*; Bruker, 2005 ▶) *T*_min_ = 0.794, *T*_max_ = 0.856
4633 measured reflections
1742 independent reflections
1214 reflections with *I* > 2σ(*I*)
*R*_int_ = 0.042

#### Refinement

*R*[*F* ^2^ > 2σ(*F* ^2^)] = 0.041
*wR*(*F* ^2^) = 0.101
*S* = 1.02
1742 reflections
101 parameters
H-atom parameters constrained
Δρ_max_ = 0.37 e Å^−3^
Δρ_min_ = −0.43 e Å^−3^

### 

Data collection: *APEX2* (Bruker, 2005 ▶); cell refinement: *SAINT-Plus* (Bruker, 2005 ▶); data reduction: *SAINT-Plus*; program(s) used to solve structure: *SHELXS97* (Sheldrick, 2008 ▶); program(s) used to refine structure: *SHELXL97* (Sheldrick, 2008 ▶); molecular graphics: *SHELXTL* (Sheldrick, 2008 ▶); software used to prepare material for publication: *SHELXTL*.

## Supplementary Material

Crystal structure: contains datablocks I, global. DOI: 10.1107/S1600536809045589/rk2177sup1.cif

Structure factors: contains datablocks I. DOI: 10.1107/S1600536809045589/rk2177Isup2.hkl

Additional supplementary materials:  crystallographic information; 3D view; checkCIF report

## Figures and Tables

**Table 1:** Hydrogen-bond geometry (Å, °)

*D*—H⋯*A*	*D*—H	H⋯*A*	*D*⋯*A*	*D*—H⋯*A*
O3—H3⋯O2^i^	0.85	1.82	2.664 (3)	170
N2—H1*A*⋯O1^ii^	0.86	2.08	2.822 (4)	145
N2—H1*B*⋯O2^iii^	0.86	2.00	2.844 (4)	169

Symmetry codes: (i) 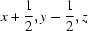; (ii) 
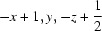
; (iii) 
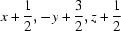
.

**Fig. 1.**
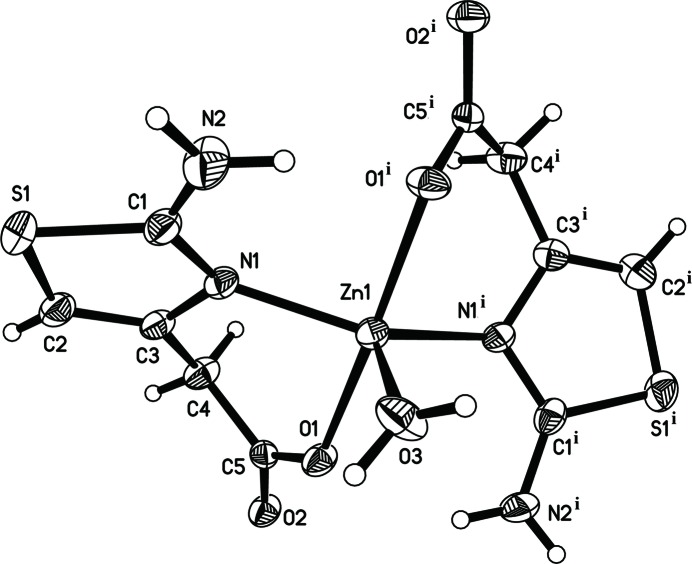
View of title molecular complex with the atom-numbering scheme. Displacement ellipsoids are drawn at the 30% probability level. H atoms are presented as a small spheres of arbitrary radius. Symmetry codes: (i) -x+1, y, -z+1/2.

**Fig. 2.**
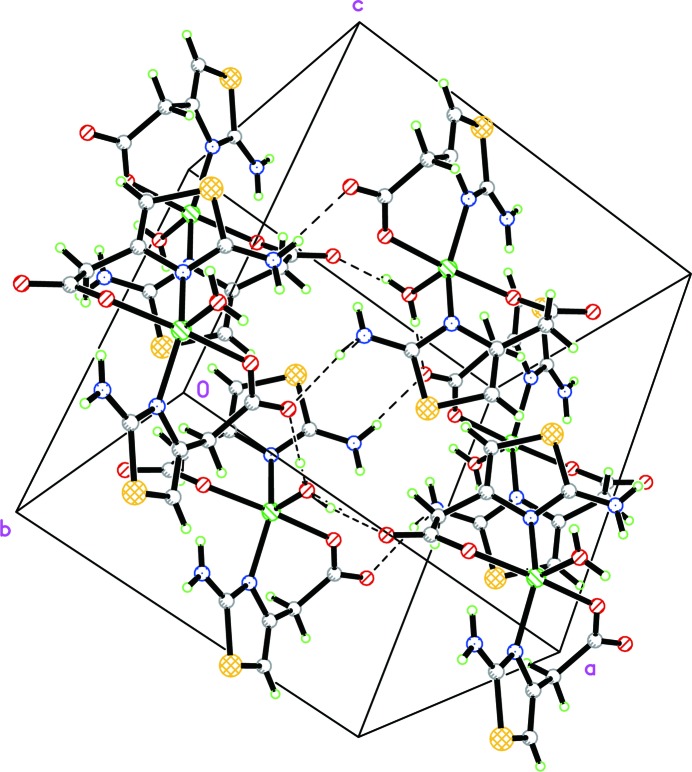
The crystal packing of I, showing formation of the three-dimensional network structure via the intermolecular O–H···O and N–H···O hydrogen bonds as denoted with dashed lines. All other hydrogen atoms were omitted for clarity.

## supplementary crystallographic information

### Comment

Some potential metal-based drugs consisting of the thiazole ligands and some physiologically active metal ions are attracting more and more attention due to their potentially higher pharmacological activity than pure thiazole ligands (Addison *et al.*, 1984; Bolos *et al.*, 1999; Chang *et al.*, 1982; Dea *et al.*, 2008). Recently, we also made our efforts to synthesize such a class of complexes and have obtained two single crystals containing 1,3-thiazole ring (Zhang *et al.* 2008a,b). The evident coordination activity of ethyl 2-aminothiazole-4-acetate (*EATA*) has been shown using AgNO_3_ as metal salt because colourless crystals were obtained in high yield overnight even at room temperature. Herein, a new five-coordinated title complex Zn(C_5_H_5_N_2_O_2_S)_2_(H_2_O), I, was synthesized using *EATA* and ZnSO_4_ as starting materials under the aid of ultrasonic irradiation. The 2-amino-4-thiazole acetate (*ATA*) ligand in complex I possibly formed *in situ* by acidic hydrolysis of *EATA* under ultrasonic irradiation because the ethanol/water solution of *EATA* is normally slightly acidic due to the present of Zn^2+^ solution.

The resulting Zn complex is built up from distorted square-pyramidal N2O2+O units (Sen *et al.* 1997), the central Zn atom is five-coordinated by two N and three O atoms [Zn–N = 2.047 (3)Å; Zn–O = 2.099 (2)Å and 1.974 (4)Å]. Besides one O atom from water molecule, two *ATA* ligands provide two N and two O atoms as coordinated atoms (Fig. 1). In the crystal structure, the intermolecular O–H···O and N–H···O hydrogen bonds (Table 1) connect these molecules into a infinite three-dimensional framework (Fig. 2).

### Experimental

The ethyl 2-aminothiazole-4-acetate (*EATA*) (1 mmol, 0.186 g) was dissolved in 5 ml of ethanol under magnetic stirring, followed by addition of 5 ml of distilled water. Then, ZnSO_4_ (1 mmol, 0.170 g) was added and dissolved after a 10-minutes ultrasonic treatment. The resulting pale-yellow solution was filtered and stayed at room temperature for half a month. Large amounts of colourless block single crystals were obtained in about 40% yield (based on Zn).

### Refinement

All hydrogen atoms attached on C, N and O atoms have been refined in the riding mode on their carrier atom, with C–H = 0.93-0.97Å, N–H = 0.86Å, O–H = 0.85Å and *U*_iso_(H) = 1.2*U*_eq_(C, N) or *U*_iso_(H) = 1.5*U*_eq_(O).

### Crystal data

**Table:**

[Zn(C_5_H_5_N_2_O_2_S)_2_(H_2_O)]	*F*(000) = 808
*M**_r_* = 397.77	*D*_x_ = 1.826 Mg m^−^^3^
Monoclinic, *C*2/*c*	Mo *K*α radiation, λ = 0.71073 Å
Hall symbol: -C 2yc	Cell parameters from 1742 reflections
*a* = 11.715 (2) Å	θ = 2.7–25.5°
*b* = 9.822 (2) Å	µ = 2.01 mm^−^^1^
*c* = 12.580 (3) Å	*T* = 295 K
β = 91.24 (3)°	Block, colourless
*V* = 1447.2 (5) Å^3^	0.12 × 0.10 × 0.08 mm
*Z* = 4	

### Data collection

**Table:**

Bruker APEXII CCD area-detector diffractometer	1742 independent reflections
Radiation source: fine-focus sealed tube	1214 reflections with *I* > 2σ(*I*)
graphite	*R*_int_ = 0.042
φ and ω scans	θ_max_ = 28.3°, θ_min_ = 2.7°
Absorption correction: multi-scan (*SADABS*; Bruker, 2005)	*h* = −15→9
*T*_min_ = 0.794, *T*_max_ = 0.856	*k* = −10→12
4633 measured reflections	*l* = −16→16

### Refinement

**Table:**

Refinement on *F*^2^	Primary atom site location: structure-invariant direct methods
Least-squares matrix: full	Secondary atom site location: difference Fourier map
*R*[*F*^2^ > 2σ(*F*^2^)] = 0.041	Hydrogen site location: inferred from neighbouring sites
*wR*(*F*^2^) = 0.101	H-atom parameters constrained
*S* = 1.02	*w* = 1/[σ^2^(*F*_o_^2^) + (0.046*P*)^2^] where *P* = (*F*_o_^2^ + 2*F*_c_^2^)/3
1742 reflections	(Δ/σ)_max_ < 0.001
101 parameters	Δρ_max_ = 0.37 e Å^−^^3^
0 restraints	Δρ_min_ = −0.42 e Å^−^^3^

### Special details

**Table:**

Geometry. All s.u.'s (except the s.u. in the dihedral angle between two l.s. planes) are estimated using the full covariance matrix. The cell s.u.'s are taken into account individually in the estimation of s.u.'s in distances, angles and torsion angles; correlations between s.u.'s in cell parameters are only used when they are defined by crystal symmetry. An approximate (isotropic) treatment of cell s.u.'s is used for estimating s.u.'s involving l.s. planes.
Refinement. Refinement of *F*^2^ against ALL reflections. The weighted *R*-factor *wR* and goodness of fit *S* are based on *F*^2^, conventional *R*-factors *R* are based on *F*, with *F* set to zero for negative *F*^2^. The threshold expression of *F*^2^ > σ(*F*^2^) is used only for calculating *R*-factors(gt) *etc*. and is not relevant to the choice of reflections for refinement. *R*-factors based on *F*^2^ are statistically about twice as large as those based on *F*, and *R*-factors based on ALL data will be even larger.

### Fractional atomic coordinates and isotropic or equivalent isotropic displacement parameters (Å^2^)

**Table:**

	*x*	*y*	*z*	*U*_iso_*/*U*_eq_	
Zn1	0.5000	0.77073 (6)	0.2500	0.03185 (19)	
S1	0.42899 (9)	0.81081 (12)	0.60112 (7)	0.0498 (3)	
O1	0.32429 (19)	0.7822 (3)	0.21472 (18)	0.0392 (6)	
O2	0.16766 (19)	0.9045 (3)	0.19131 (17)	0.0411 (6)	
O3	0.5000	0.5698 (4)	0.2500	0.0499 (10)	
H3	0.5551	0.5241	0.2253	0.075*	
N1	0.4607 (2)	0.8338 (3)	0.3999 (2)	0.0329 (7)	
N2	0.6077 (3)	0.7192 (4)	0.4935 (2)	0.0536 (9)	
H1A	0.6459	0.7073	0.4366	0.064*	
H1B	0.6340	0.6893	0.5534	0.064*	
C5	0.2579 (3)	0.8773 (4)	0.2413 (2)	0.0302 (8)	
C1	0.5089 (3)	0.7835 (4)	0.4888 (3)	0.0382 (8)	
C3	0.3572 (3)	0.8988 (4)	0.4224 (3)	0.0349 (8)	
C2	0.3275 (3)	0.8960 (4)	0.5241 (3)	0.0432 (9)	
H2	0.2612	0.9346	0.5502	0.052*	
C4	0.2909 (3)	0.9663 (4)	0.3346 (3)	0.0417 (9)	
H4A	0.3355	1.0420	0.3087	0.050*	
H4B	0.2217	1.0037	0.3640	0.050*	

### Atomic displacement parameters (Å^2^)

**Table:**

	*U*^11^	*U*^22^	*U*^33^	*U*^12^	*U*^13^	*U*^23^
Zn1	0.0295 (3)	0.0373 (4)	0.0286 (3)	0.000	−0.0023 (2)	0.000
S1	0.0541 (7)	0.0690 (8)	0.0262 (5)	−0.0060 (5)	−0.0011 (4)	0.0010 (4)
O1	0.0309 (13)	0.0479 (16)	0.0384 (14)	0.0085 (11)	−0.0078 (11)	−0.0132 (12)
O2	0.0339 (14)	0.0525 (17)	0.0365 (14)	0.0091 (11)	−0.0070 (11)	−0.0076 (12)
O3	0.031 (2)	0.034 (2)	0.085 (3)	0.000	0.0120 (18)	0.000
N1	0.0329 (16)	0.0388 (18)	0.0266 (14)	0.0008 (13)	−0.0058 (12)	0.0007 (12)
N2	0.049 (2)	0.075 (3)	0.0363 (18)	0.0228 (18)	−0.0075 (15)	0.0126 (17)
C5	0.0248 (17)	0.039 (2)	0.0272 (17)	−0.0004 (14)	−0.0009 (13)	0.0011 (14)
C1	0.044 (2)	0.043 (2)	0.0274 (18)	−0.0075 (17)	−0.0048 (15)	0.0016 (15)
C3	0.0353 (19)	0.036 (2)	0.0331 (19)	−0.0005 (15)	−0.0062 (14)	−0.0086 (15)
C2	0.039 (2)	0.054 (3)	0.037 (2)	−0.0001 (18)	−0.0008 (16)	−0.0158 (17)
C4	0.041 (2)	0.042 (2)	0.042 (2)	0.0084 (16)	−0.0086 (16)	−0.0108 (17)

### Geometric parameters (Å, °)

**Table:**

Zn1—O3	1.974 (4)	N1—C3	1.404 (4)
Zn1—N1	2.047 (3)	N2—C1	1.319 (5)
Zn1—N1^i^	2.047 (3)	N2—H1A	0.8600
Zn1—O1^i^	2.099 (2)	N2—H1B	0.8600
Zn1—O1	2.099 (2)	C5—C4	1.507 (5)
S1—C1	1.733 (4)	C3—C2	1.335 (4)
S1—C2	1.733 (4)	C3—C4	1.492 (5)
O1—C5	1.266 (4)	C2—H2	0.9300
O2—C5	1.247 (3)	C4—H4A	0.9700
O3—H3	0.8500	C4—H4B	0.9700
N1—C1	1.336 (4)		
			
O3—Zn1—N1	107.61 (8)	H1A—N2—H1B	120.0
O3—Zn1—N1^i^	107.61 (8)	O2—C5—O1	122.9 (3)
N1—Zn1—N1^i^	144.78 (17)	O2—C5—C4	118.0 (3)
O3—Zn1—O1^i^	93.07 (7)	O1—C5—C4	119.0 (3)
N1—Zn1—O1^i^	91.60 (10)	N2—C1—N1	124.7 (3)
N1^i^—Zn1—O1^i^	86.54 (10)	N2—C1—S1	121.8 (3)
O3—Zn1—O1	93.07 (7)	N1—C1—S1	113.6 (3)
N1—Zn1—O1	86.54 (10)	C2—C3—N1	115.4 (3)
N1^i^—Zn1—O1	91.60 (10)	C2—C3—C4	125.2 (3)
O1^i^—Zn1—O1	173.87 (14)	N1—C3—C4	119.5 (3)
C1—S1—C2	89.73 (17)	C3—C2—S1	110.8 (3)
C5—O1—Zn1	126.1 (2)	C3—C2—H2	124.6
Zn1—O3—H3	121.8	S1—C2—H2	124.6
H3—O3—H3^i^	116.3	C3—C4—C5	116.1 (3)
C1—N1—C3	110.5 (3)	C3—C4—H4A	108.3
C1—N1—Zn1	124.0 (3)	C5—C4—H4A	108.3
C3—N1—Zn1	122.4 (2)	C3—C4—H4B	108.3
C1—N2—H1A	120.0	C5—C4—H4B	108.3
C1—N2—H1B	120.0	H4A—C4—H4B	107.4

Symmetry codes: (i) −*x*+1, *y*, −*z*+1/2.

### Hydrogen-bond geometry (Å, °)

**Table:**

*D*—H···*A*	*D*—H	H···*A*	*D*···*A*	*D*—H···*A*
O3—H3···O2^ii^	0.85	1.82	2.664 (3)	170
N2—H1A···O1^i^	0.86	2.08	2.822 (4)	145
N2—H1B···O2^iii^	0.86	2.00	2.844 (4)	169

Symmetry codes: (ii) *x*+1/2, *y*−1/2, *z*; (i) −*x*+1, *y*, −*z*+1/2; (iii) *x*+1/2, −*y*+3/2, *z*+1/2.
